# Long-term intensive endurance exercise training is associated to reduced markers of cellular senescence in the colon mucosa of older adults

**DOI:** 10.1038/s41514-023-00100-w

**Published:** 2023-02-27

**Authors:** Marco Demaria, Beatrice Bertozzi, Nicola Veronese, Francesco Spelta, Edda Cava, Valeria Tosti, Laura Piccio, Dayna S. Early, Luigi Fontana

**Affiliations:** 1 European Research Institute for the Biology of Ageing (ERIBA), University Medical Center Groningen (UMCG), University of Groningen (RUG), Groningen, Netherlands; 2 Department of Medicine, Washington University School of Medicine, St. Louis, MO USA; 3 Geriatric Unit, Department of Internal Medicine and Geriatrics, University of Palermo, Palermo, Italy; 4 Geriatric Unit, AULSS 9 Scaligera, “Mater Salutis” Hospital, Legnago, Verona, Italy; 5 Unit of Dietetic and Clinical Nutrition, San Camillo, Forlanini Hospital, Rome, Italy; 6 Department of Neurology, Washington University, St.Louis, MO USA; 7 Brain and Mind Centre, University of Sydney, Sydney, NSW Australia; 8 Charles Perkins Centre, Faculty of Medicine and Health, University of Sydney, Sydney, NSW 2006 Australia; 9 Department of Endocrinology, Royal Prince Alfred Hospital, Sydney, NSW 2006 Australia; 10 Department of Clinical and Experimental Sciences, Brescia University School of Medicine, Brescia, Italy

**Keywords:** Senescence, Biomarkers

## Abstract

Regular endurance exercise training is an effective intervention for the maintenance of metabolic health and the prevention of many age-associated chronic diseases. Several metabolic and inflammatory factors are involved in the health-promoting effects of exercise training, but regulatory mechanisms remain poorly understood. Cellular senescence—a state of irreversible growth arrest—is considered a basic mechanism of aging. Senescent cells accumulate over time and promote a variety of age-related pathologies from neurodegenerative disorders to cancer. Whether long-term intensive exercise training affect the accumulation of age-associated cellular senescence is still unclear. Here, we show that the classical senescence markers p16 and IL-6 were markedly higher in the colon mucosa of middle-aged and older overweight adults than in young sedentary individuals, but this upregulation was significantly blunted in age-matched endurance runners. Interestingly, we observe a linear correlation between the level of p16 and the triglycerides to HDL ratio, a marker of colon adenoma risk and cardiometabolic dysfunction. Our data suggest that chronic high-volume high-intensity endurance exercise can play a role in preventing the accumulation of senescent cells in cancer-prone tissues like colon mucosa with age. Future studies are warranted to elucidate if other tissues are also affected, and what are the molecular and cellular mechanisms that mediate the senopreventative effects of different forms of exercise training.

Regular physical exercise is one of the key pillars for health promotion since ancient times, although our ancestors did not know the biological processes responsible for its beneficial effects. Data from animal and human randomized trials indicate that aerobic exercise training improves glucose tolerance, insulin sensitivity and lipid metabolism through multiple mechanisms, including mitochondrial biogenesis, increased expression of the insulin responsive glucose transporter type 4 (GLUT4) and lipoprotein lipase in the skeletal muscle^1,2^. Regular exercise training also promotes visceral fat loss, reduces inflammation and oxidative stress, and improves left ventricular diastolic function in overweight men and women^1,3–6^.

Accumulating data show that physical activity evokes profound metabolic and molecular responses not only in key metabolic organs (skeletal muscle, adipose tissues, and liver), but also in tissues at high risk of neoplastic transformation. Epidemiological studies suggest an inverse association between physical activity and risk for 13 different types of cancer, in particular for colon and breast cancer^7–9^. Regular exercise training can also improve prognosis among breast and colorectal cancer survivors^10,11^ by long-term regulation of various metabolic, inflammatory and aging pathways that promote DNA and cellular repair, proteostasis, replicative stress resistance, and apoptosis of permanently damaged cells^12^.

One of the fundamental cellular mechanisms regulating aging and tumor development is cellular senescence^13^. Cellular senescence is a state of irreversible proliferative arrest triggered by diverse DNA or mitochondrial damages to prevent propagation of damaged cells^14^. Senescent cells are characterized by the engagement of the Cyclin-dependent kinase inhibitors p16^*Ink4a*^ (p16) and p21^CIP1^ (p21)^15^, enhanced lysosomal activity, and a hypersecretory phenotype known as Senescence-Associated Secretory Phenotype (SASP). The SASP remain highly heterogeneous and dependent on various intrinsic and extrinsic factors^16,17^. However, persistent senescent cells can cause chronic low-level inflammation and aberrant tissue growth and remodeling via SASP factors^18–23^.

Lifestyle factors can have consequences on induction and accumulation of cellular senescence. For example, caloric restriction (CR), a well-known and highly conserved anti-aging and anti-cancer intervention, is associated to reduced accumulation of senescent cells in both mice and humans^24,25^. Interestingly, we have also recently shown that, at least in mice, high-protein and high-fat diets lead to premature hepatic accumulation of hyper-inflammatory senescent cells^26^. Besides dietary approaches, it has been shown that a 12-week exercise program reduces circulating senescence biomarkers in older adults^27^ and a recent human study suggests that the number of senescent cells of the adipose tissue is inversely correlated to physical function in older women^28^. However, whether regular vigorous aerobic exercise can prevent accumulation of age-associated senescence, especially in highly proliferating cancer prone tissues, remains controversial^29^. Here, we studied the effects of chronic intensive endurance exercise training on cardiometabolic health and candidate biomarkers of cell senescence in colon mucosa biopsies of master athletes who ran an average 48 miles/week (range 30 to 90 miles/week) for an average of 21 years (range 5–35 years).

Participants in this study were endurance runners (mean age 57 ± 10 years) consuming usual American diets (EX); age- and sex-matched sedentary (regular exercise < 1 h per week) controls eating Western diets (WD-o); and very young (mean age 24.3 ± 2 years) sedentary controls (WD-y) who should have negligible numbers of senescent cells. Average calorie intake in the EX group was 2806 ± 618 kcal/day, 13 and 7% higher than in the WD-o (2443 ± 407 kcal/day) and WD-y (2618 ± 712 kcal/day) groups, respectively (*p* < 0.05 for EX vs. WD-o). The percentages of total energy intake derived from protein, carbohydrate, and fat were similar among the groups: 15.7%, 51.8%, and 32.5%, respectively, in EX; 15.1%, 50.4%, and 32.8%, in WD-o; 17.2%, 48.2%, and 33.4% in WD-y.

In Table 1, we reported the study sample’s summary statistics, including the distribution of age, sex, body mass index, DXA body fat percentage and lean mass, and a range of fitness and cardiometabolic parameters. BMI, body fat, resting heart rate, LDLc, total cholesterol HDL ratio, triglycerides, triglycerides to HDL ratio, fasting glucose, fasting insulin, HOMA-IR, and total white blood cell count were significantly lower in the EX group than in the WD-o group (*p* < 0.05). As expected, EX volunteers had significantly higher VO_2_max and HDLc than WD-o participants (*p* < 0.05).

**Table 1:** Characteristics of the study subjects.

	EX group	WD-o group	WD-y group	Among group P
	(*n* = 44)	(*n* = 44)	(*n* = 6)	
Age (years)	57 ± 10	57 ± 9	24.3 ± 2^a,c^	<0.001
Sex (M:F)	37:7	37:7	4:2	–
Height (m)	1.75 ± 0.1	1.76 ± 0.1^a^	1.79 ± 0.1	NS
Weight (Kg)	70.0 ± 10	78.8 ± 14^a^	82.6 ± 13^b^	<0.001
BMI (Kg/m^2^)	22.7 ± 4	25.3 ± 2.7^a^	25.7 ± 1^b^	<0.001
Body fat (% body weight)	14.8 ± 6.5	25.2 ± 6.6^a^	17.9 ± 8.2^d^	<0.001
Lean mass (kg)	56.1 ± 8	54.7 ± 11	63.3 ± 14	NS
Resting heart rate (b/min)	52 ± 8	63 ± 10^a^	66 ± 12^b^	<0.001
VO2max (ml/Kg/min)	51 ± 10	33 ± 7^a^	–	<0.001
SBP (mm Hg)	126 ± 19	129 ± 14	129 ± 11	NS
DBP (mm Hg)	73 ± 10	79 ± 9^b^	78 ± 11	0.055
LDL-c (mg/dl)	92 ± 22	115 ± 28^a^	94 ± 24	0.004
HDL-c (mg/dl)	68 ± 17	55 ± 15^a^	64 ± 18	<0.001
Triglycerides (mg/dl)	64 ± 22	120 ± 71^a^	61 ± 33^d^	<0.001
TChol/HDL ratio	2.6 ± 0.5	3.8 ± 1.0^a^	2.9 ± 0.9^d^	<0.001
TG/HDL ratio	1 ± 0.4	2.5 ± 2.0^a^	0.9 ± 0.6^d^	<0.001
Fasting glucose (mg/dl)	90 ± 8	94 ± 9^b^	82 ± 5^c^	0.001
Fasting insulin (mg/dl)	3.0 ± 2.3	7.6 ± 5.4^a^	6.6 ± 3	<0.001
HOMA-IR	0.7 ± 0.6	1.8 ± 1.3^a^	1.3 ± 0.6	<0.001
WBC (K/cumm)	4.5 ± 1.2	5.8 ± 1.6^a^	4.9 ± 0.5	<0.001
hsCRP (mg/L)	0.7 ± 0.6	1.8 ± 1.3	0.8 ± 0.3	NS

All values are means ± SD.

Significantly different from EX group, ^a^*P* ≤ 0.003, ^b^*P* ≤ 0.05.

Significantly different from WD-o group, ^c^*P* ≤ 0.003, ^d^*P* ≤ 0.05.

To investigate the effects of long-term EX on biomarkers of cell senescence, we collected colon mucosa biopsies in a subset of 11 middle-aged (58.6 ± 8.3 years), weight-stable and lean (BMI, 24.5 ± 2.8 kg/m^2^) master athletes, 10 age- and sex-matched nonobese (BMI, 27.1 ± 2.3 kg/m^2^) and 6 sedentary young (24.3 ± 2 years) and lean (BMI, 25.7 ± 1 kg/m^2^) control subjects (Supplementary table 1). Because p16 is still considered one of the most relevant cell senescence markers in human specimens, and because p16 measurements were included in most of the previous studies on the effect of physical exercise on senescence markers, we measured its mRNA abundance. As expected, p16 levels were markedly higher in older than younger sedentary individuals consuming Western diets (Fig. 1A). Strikingly, this upregulation was significantly blunted in endurance runners (Fig. 1A). p21 is another important regulator of cell cycle arrest often dysregulated during senescence. Similar to what observed for p16, p21 levels were upregulated in older sedentary individuals compared to young sedentary or endurance runners (Fig. 1B). However, the difference in expression between groups did not reach statistical significance. The detrimental functions of cellular senescence are, at least partly, mediated by pro-inflammatory secreted factors. The composition of pro-inflammatory SASP is variable, but IL-6 remains one of the most consistent SASP factors^13^. In accordance, IL-6 mRNA levels of colon mucosa were significantly higher in old sedentary individuals consuming Western diet, whereas IL-6 levels in master athletes where low and similar to those of very young sedentary people consuming Western diets (Fig. 1C). We then measured levels of two additional SASP factors, IL8 and MMP3. We observed a trend for the upregulation of MMP3 and IL8 in older sedentary individuals compared to young sedentary and endurance runners, but there was no statistical significance (Fig. 1D, E). p16 mRNA levels correlated linearly with p21 (*r* = 0.758; *p* < 0.001) and IL-6 (*r* = 0.798; *p* < 0.001) mRNA levels (not shown). To evaluate potential links between the senescence burden and metabolic alterations, we then studied the association of p16 levels with various metabolic parameters. Strikingly, p16 mRNA levels were linearly correlated with the triglycerides to HDL ratio, a well-accepted marker of metabolic syndrome, coronary heart disease and colon adenoma risk (Fig. 1F)^30–32^. Interestingly, in patients with early-stage colorectal cancer, the combination of obesity and low HDL-cholesterol and high triglycerides levels predicts worst cancer survival^33^.

**Fig. 1:**
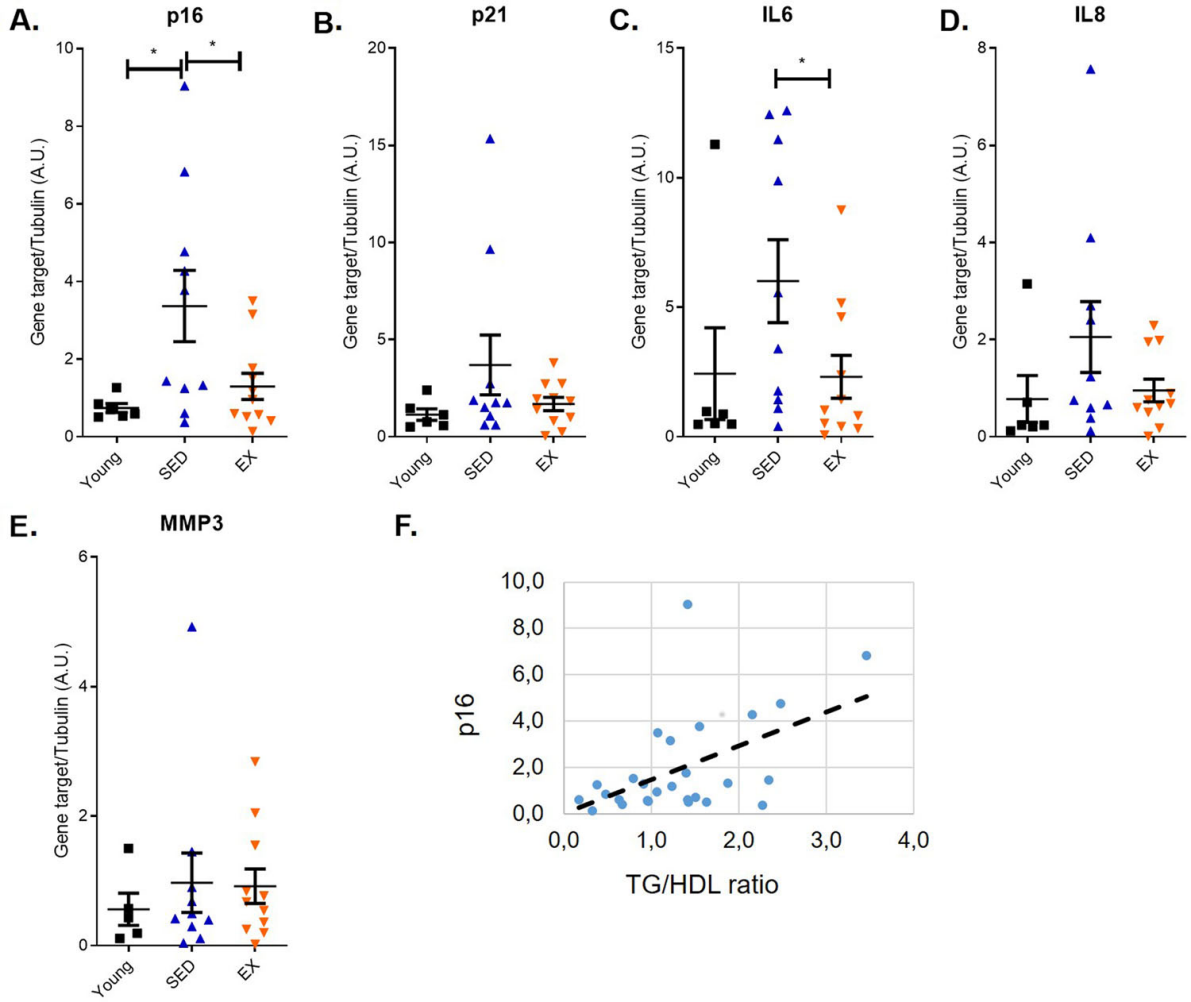
Expression of senescence-associated genes in the colon mucosa of master athletes and sedentary controls. RNA was extracted from the sigmoid portion of the colon of human volunteers. The groups were: EX, exercised volunteers of average age 57 ± 10 years; age-matched sedentary controls (SED); young, volunteers of average age 24.3 ± 2 years. mRNA encoding p16 (**A**), p21 (**B**), IL6 (**C**), IL8 (**D**), and MMP3 (**E**) were quantified by qRT-PCR. mRNA encoding tubulin was used as internal control (*N* = 5–11 with each sample indicated by an individual dot). Panel **F** shows the relationship between p16 mRNA levels and the triglycerides to HDL ratio. All values are represented together with means and SEM. One-way Anova, *p < 0.05.

Our results are preliminary and limited, in particular in light of characterizing senescence-associated phenotypes and the type of cells more affected by these changes. Nevertheless, the findings shown here suggest that chronic high-volume high-intensity, unlike low-volume^34^, endurance exercise can play a major role in preventing the accumulation of senescent cells in cancer prone tissues like colon mucosa with age. This is important because data from transgenic mouse models, including our p16-3MR mouse^19^, have shown that ablation of senescent cells is sufficient to systemically reduce inflammation, rejuvenate tissue functions, alleviate various age-related conditions, improve health and extend longevity^13^. As senescent cells are likely contributor to dysregulated inflammatory responses, our data are in line with a previous report showing that the level of stress-induced (acute exercise) inflammatory markers is reduced in muscle and blood of lifelong aerobic exercising older men compared to old healthy nonexercisers^35^. In addition, prevention of cell senescence could partly explain the anti-cancer effect of lifelong aerobic exercise^36^. Future studies should be focusing on understanding which tissues are most affected, and what are the molecular and cellular mechanisms that mediate the senopreventative effect of endurance exercise training. Moreover, it will be key to analyze individuals following different physical exercise regimens, including resistance and high-intensity interval training.

## Methods

### Patients and tissue collection

This study sample includes three groups of volunteers, named from hereafter EX, WD-o, and WD-y. The EX group consisted of 44 master athletes who ran at least 30 miles/week (range 30–90 miles/week) or expended similar amount of energy by cycling or swimming, for at least the previous three years (range 3–35 years). The control (WD-o) group comprised 44 age- and sex-matched individuals reporting less than 1 hour of physical activity per week, recruited from the St. Louis metropolitan area. A third control group with negligible numbers of senescent cells consisted of 6 very young (mean age 24.3 ± 2 years) sedentary controls (WD-y) consuming Western diets. All the participants reported weight stability, defined as less than a 2-kg change in body weight in the preceding 6 months. Participants recorded all food and beverage intake for 7 consecutive days. Food records were analyzed by our dietitian by using the NDS-R pro-gram (v.4.03_31) and used to define the western diet consumers. None of the participants had evidence of chronic disease, smoked cigarettes, or took medications that could affect the outcome variables. The present study (HRPO #: 01-0804) was approved by the Human Studies Committee of Washington University School of Medicine, and all subjects gave written informed consent before their participation. Height and body weight were obtained in the morning after an overnight fast, with the participants wearing only underwear and a hospital gown. Total body fat mass and fat-free mass were determined by dual-energy X-ray absorptiometry (DXA; QDR 1000/w; Hologic). VO2max was determined by indirect calorimetry during an incremental exercise test to exhaustion^37^. Participants walked on a level treadmill at a pace that elicited 60–70% of age-predicted maximal heart rate for a 5-minute warm-up. The speed was then set at the fastest comfortable pace, and the grade was increased 1–2% every 1–2 minutes until volitional exhaustion, electrocardiographic changes, or other abnormalities that rendered it unsafe to continue. Blood pressure was measured with an oscillometric blood pressure monitor (Dinamap Procare 200; GE Healthcare, Waukesha, WI) in the morning after a 12-h fast. In the EX group, blood pressure was measured at least 48 h after the last exercise session. A venous blood sample was taken to determine lipid and hormone concentrations after subjects had fasted overnight. In the EX group, blood samples were obtained ≥48 h after the last exercise session. Measurement of serum lipid and lipoprotein-cholesterol concentrations, glucose, insulin, C-reactive protein was performed in the Barnes-Jewish Hospital Laboratory by automated enzymatic, radioimmunoassay and ELISA commercial kits. Insulin resistance was calculated using homeostasis model assessment of insulin resistance 9HOMA-IR = [fasting glucose (mmol/l) × fasting insulin 59]/22.5).

### RNA isolation and cDNA synthesis

Biopsy specimens of normal-appearing sigmoidal colon mucosa were collected from a subset of 11 EX, 11 WD-o, and 5 WD-y volunteers in the morning after an overnight fast and a preparation with an enema containing water. Colonic mucosal specimens were immediately washed in PBS and then flash-frozen in liquid nitrogen and stored at −80 °C until processed. Tissues were homogenized in liquid nitrogen. For each sample, 20 mg of tissue powder was used to isolate total RNA using the Isolate II RNA Mini Kit (Bioline). In all, 250–500 ng of RNA was reverse transcribed into cDNA using a kit (Applied Biosystems).

### Real time-qPCR

qRT-PCR reactions were performed with the LightCycler 480 Instrument II (Roche) using UPL system (Roche) with a SensiFast Probe kit (Bioline). The reactions were carried out in a total volume of 10 *μ*l using a TaqMan assay. Tubulin was used for normalization of the CT values. List of primers/probe combination:

Tubulin: FW- cttcgtctccgccatcag; RV-cgtgttccaggcagtagagc; Probe #40

Cdkn2a (p16): FW-gagcagcatggagcctc; RV-cgtaactattcggtgcgttg; Probe #67

Cdkn1a (p21): FW-tcactgtcttgtacccttgtgc; RV-ggcgtttggagtggtagaaa; Probe #32

IL6: FW-caggagcccagctatgaact; RV-gaaggcagcaggcaacac; Probe #45

IL8: FW-gagcactccataaggcacaaa; RV-atggttccttccggtggt; Probe #72

MMP3: FW-caaaacatatttctttgtagaggacaa; RV-ttcagctatttgcttgggaaa; Probe #36

### Statistical analysis

One-way analysis of variance (ANOVA) was used to compare group variables, followed by Tukey post-hoc testing when indicated. One-way ANOVA with Games-Howell was performed for distributions where equal variances could not be assumed. Pearson correlation was used to assess associations between continuous variables. Statistical significance was set at *P* < 0.05 for all tests. All data were analyzed by using SPSS software, version 28.0 (SPSS Inc, Chicago). Data are expressed as mean ± SEM or SD (indicated). A difference with *P*-values < 0.05 were considered statistically significant.

## Supplementary information

Supplementary table 1

## Data Availability

Data are available from the corresponding authors upon reasonable request.
