# Supplementary material for: Long-term intensive endurance exercise training is associated to reduced markers of cellular senescence in the colon mucosa of older adults
Source: NPJ Aging. 2023 Feb 27;9(1):3. doi: 10.1038/s41514-023-00100-w (PMC9971019; doi:10.1038/s41514-023-00100-w)
Supplement: Supplementary file 1 — Supplementary table 1 [file 41514_2023_100_MOESM1_ESM.docx]

**Supplementary Table 1**. Characteristics of the subjects who underwent sigmoidoscopy

|  | **EX group**  **(n=11)** | **WD-o group**  **(n=10)** | **WD-y group**  **(n=6)** | **Among**  **group P** |
| --- | --- | --- | --- | --- |
| **Age (yrs)** | 58.6±8.3 | 63.2±7 | 24.3±2^1,3^ | <0.001 |
| **Sex (M:F)** | 9:2 | 8:2 | 4:2 |  |
| **Height (m)** | 1.77±0.1 | 1.76±0.1 | 1.79±0.1 | NS |
| **Weight (Kg)** | 76.7±11 | 83.5±8 | 82.6±13 | NS |
| **BMI (Kg/m^2^)** | 24.5±2.8 | 27.1±2.3^2^ | 25.7±1 | 0.05 |
| **Body fat (% body weight)** | 19.4±5 | 28.9±9^2^ | 17.9±8^3^ | 0.008 |
| **Lean mass (kg)** | 57.8±9 | 55.9±10 | 63.3±14 | NS |
| **Resting heart rate (b/min)** | 55±10 | 69±10^2^ | 66±12 | 0.01 |
| **SBP (mm Hg)** | 129±17 | 131±16 | 129±11 | NS |
| **DBP (mm Hg)** | 78±10 | 77±10 | 78±11 | NS |
| **LDL-c (mg/dl)** | 95±23 | 110±29 | 94±24 | NS |
| **HDL-c (mg/dl)** | 72±19 | 59±21 | 64±18 | NS |
| **Triglycerides (mg/dl)** | 66±22 | 104±37^2^ | 61±33^3^ | 0.01 |
| **TChol/HDL ratio** | 2.6±0.5 | 3.4±0.7 | 2.9±0.9 | 0.06 |
| **TG/HDL ratio** | 1.0±0.3 | 1.9±0.8^1^ | 0.9±0.6^3^ | 0.002 |
| **Fasting glucose (mg/dl)** | 90±9 | 97±13 | 82±5^3^ | 0.05 |
| **Fasting insulin (mg/dl)** | 5.4±2.5 | 10.3±5.2^2^ | 6.6±3 | 0.02 |
| **HOMA-IR** | 1.2±0.7 | 2.5±1.2^2^ | 1.3±0.6 | 0.01 |
| **WBC (K/cumm)** | 5.0±1.6 | 6.0±1.7^1^ | 4.9±0.5 | <0.001 |
| **hsCRP (mg/L)** | 1.3±1.1 | 1.9±1.6 | 0.8±0.3 | NS |

All values are means ± SD.

Significantly different from EX group, P≤0.004^1^, P≤0.05^2^

Significantly different from WD-o group, P≤0.05^3^
